# Theoretical Calculation of the Gas-Sensing Properties of Pt-Decorated Carbon Nanotubes

**DOI:** 10.3390/s131115159

**Published:** 2013-11-06

**Authors:** Xiaoxing Zhang, Ziqiang Dai, Li Wei, Naifeng Liang, Xiaoqing Wu

**Affiliations:** 1 State Key Laboratory of Power Transmission Equipment & System Security and New Technology, Chongqing University, Shapingba District, Chongqing 400044, China; E-Mails: daizq382812@163.com (Z.D.); wufeng.200809@163.com (X.W.); 2 State Grid Xinjiang Electric Power Company, Construction Road, Urumchi 830011, China; E-Mails: liweicqu2007@hotmail.com (L.W.); Liangnaifeng@xj.sgcc.com.cn (N.L.)

**Keywords:** platinum-decorated carbon nanotubes, density functional theory, frontier orbital

## Abstract

The gas-sensing properties of Pt-decorated carbon nanotubes (CNTs), which provide a foundation for the fabrication of sensors, have been evaluated. In this study, we calculated the gas adsorption of Pt-decorated (8,0) single-wall CNTs (Pt-SWCNTs) with SO_2_, H_2_S, and CO using GGA/PW91 method based on density functional theory. The adsorption energies and the changes in geometric and electronic structures after absorption were comprehensively analyzed to estimate the responses of Pt-SWCNTs. Results indicated that Pt-SWCNTs can respond to the three gases. The electrical characteristics of Pt-SWCNTs show different changes after adsorption. Pt-SWCNTs donate electrons and increase the number of hole carriers after adsorbing SO_2_, thereby enhancing its conductivity. When H_2_S is adsorbed on CNTs, electrons are transferred from H_2_S to Pt-SWCNTs, converting Pt-SWCNTs from *p*-type to *n*-type sensors with improved conductivity. However, Pt-SWCNTs obtain electrons and show decreased conductivity when reacted with CO gas.

## 1. Introduction

Carbon nanotubes (CNTs) have structures with abundant pores, large surface-to-volume ratios, and strong adsorption and desorption capabilities for gases. Gas molecules that adsorb on the surface of CNTs change the shape of CNTs and trigger redistribution of electrons, leading to a macroscopic change in resistance [1]. Kong *et al.* [2] used chemical vapor deposition (CVD) to fabricate single-wall CNTs (SWCNTs) on SiO_2_/Si substrates to detect NO_2_ (2 ppm to 200 ppm) and NH_3_ (0.1% to 1%) diluted in air or Ar. Results showed that the conductivity of SWCNTs decreases threefold after adsorbing NH_3_, whereas the conductivity increases threefold after adsorbing NO_2_. Unlike traditional gas sensors, CNT gas sensors exhibit faster response, higher sensitivity, smaller size, and lower working temperatures [3,4]. These advantages make CNTs suitable for application in industries, the medical field, and environmental protection. Recently, the CNT gas sensor has received extensive research attention and achieved certain interesting results. It is significant to study the gas sensitive properties of CNTs, which is the foundation of sensor design. In this paper, the gas sensitive property of CNTs is studied mainly through theoretical calculation analysis. Intrinsic CNTs can only detect several strong oxidizing and reducing gases, while other gases are only weakly adsorbed with low sensitivity because of the structure and chemical properties of intrinsic CNTs [5–7]. To overcome this limitation, some researchers have proposed various physical and chemical modifications, such as introduction of new active sites on the surface of CNTs. The authors in [8] showed that polar groups (COOH, NH_2_, NO_2_ and H_2_PO_3_) are promising candidates for enhancing CO_2_ and CH_4_ adsorption capacity by strengthening adsorption and activating exposed edges and terraces to introduce additional binding sites. Peng *et al.* [9] found B-doped CNT gas sensors have a good sensitivity to CO and H_2_O. Besides, transition metals are rich in *d*-electrons and empty orbitals, wherein small gas molecules can bond strongly to the metal when adsorbed on the surface [10,11]. Studies [12] indicated that compared with intrinsic CNTs, CNTs with metal depositions have better sensitivity. For instance, Pt- and Au-functionalized CNTs are more sensitive by an order of magnitude for NO_2_ and NH_3_ detection than intrinsic CNTs. The response characteristics of sensors largely depend on the number of active sites, which can strengthen the response of sensors [11]. Pt can adsorb small molecules [13–16], and Pt has a good catalytic activity. CNTs have excellent physical and chemical properties and unique structures that can be used as a supporting material that influences the activity of Pt catalysts [17]. Conversely, the catalytic activity of Pt can improve the gas sensing properties of CNTs. Therefore the present study introduces Pt as a new active site. The adsorptive processes and properties of Pt-SWCNTs for SO_2_, H_2_S, and CO are calculated. These gases are highly toxic to the human body, making this research significant by providing theoretical support and a good foundation for the fabrication of suitable CNT-based sensors.

## 2. Computation Model and Methods

We built (8,0) SWCNTs and gas molecule models using Materials Studio (Accelrys, San Diego, CA, USA), a molecular dynamics simulation software. The geometries and properties of the system were derived using the quantum mechanics program DMol^3^ code (Accelrys). We adopted GGA to treat the electronic exchange and correlation effects, as described by PW91 [18]. Pt is a heavy metal with an atomic number of 78; therefore, DFT semi-core pseudopotentials [19] were used to manage the interactions between the nucleus and the valence electron. To ensure accuracy, the energy threshold and self-consistent field convergence criteria were set to 2.72 × 10^−4^ and 2.72 × 10^−5^ eV, respectively. The space orbital cutoff radius was set to 0.40 nm, whereas the Brillouin zone k-point sampling was performed in a 1 × 1 × 2 [20,21] Monkhorst–Pack mesh. A 2.50 nm × 2.50 nm × 0.85 nm periodic boundary was adopted to avoid the interaction between adjacent cells.

**Figure 1.**
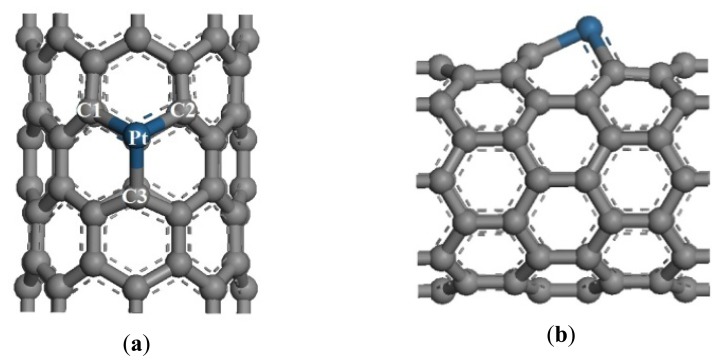
Structural model of Pt-SWCNTs. (**a**) Front view; (**b**) side view.

Two SWCNT unit cells were selected as intrinsic CNTs to build Pt-SWCNTs. According to references [11,22], Pt can easily adsorb on the vacancy defects of SWCNTs, and its adsorption energy is 6.400 eV, which is greater than that of Pt adsorbed on perfect crystal surface (2.750 eV). Accordingly, the present research selected this Pt-SWCNT model with geometry optimization structure, as shown in Figure 1.

## 3. Results and Discussion

The radius of a Pt atom is 0.183 nm, which is greater than that of a C atom (0.070 nm). Thus, Pt is highlighted on the CNT surface. The bond lengths between the Pt atom and three adjacent C atoms changed from 0.142 nm to 0.199, 0.199, and 0.189 nm for Pt-C1, Pt-C2, and Pt-C3, respectively. These results are consistent with the reference [22].

The quantum chemical energies of Pt-SWCNTs and gas molecules, as well as the optimized structure of the adsorption systems (*E_Pt-SWCNTs_*, *E_gas_*, and *E_gas-Pt-SWCNTs_*) were calculated. The adsorption energy (*E_b_*) between a gas molecule and CNTs can be calculated by the following formula:

(1) $$E_{b}=E_{\text{gas}-Pt-\text{SWCNTs}}-E_{Pt-\text{SWCNTs}}-E_{\text{gas}}$$

At *E_b_* < 0, the energy of the absorption system is less than the total energy of gas molecules and Pt-SWCNTs. Therefore, the reaction is exothermic and spontaneous. Greater adsorption energy releases more energy during the reaction process. However, when *E*_b_ > 0 it is relatively difficult for the reaction to continue because of the energy required.

In actual practice, the gas-sensitive response of the sensor is evaluated by the changes in electrical characteristics (e.g., resistance) of sensors. Therefore, we also calculated and analyzed the electronic structure of Pt-SWCNTs, gas molecules, and adsorption system. *E_HOMO_* and *E_LUMO_* represent the highest occupied molecular orbital (HOMO) energy and the lowest unoccupied orbital (LUMO) energy, respectively. *E*g is the difference of *E_LUMO_* and *E_HOMO_*, and *Q* is the net charge of the system. The parameters are defined as follows:

(2) $$E_{L-H}=\left|E_{{\text{LUMO}}^{\left(Pt-\text{SWCNTs}\right)}}-E_{{\text{HOMO}}^{\left(\text{gas}\right)}}\right|$$

(3) $$E_{H-L}=\left|E_{{\text{LUMO}}^{\left(\text{gas}\right)}}-E_{{\text{HOMO}}^{\left(Pt-\text{SWCNTs}\right)}}\right|$$

(4) $$\Delta Q_{\text{SWCNTs}}=Q_{{\text{SWCNTs}}^{\left(\text{gas}-Pt-\text{SWCNTs}\right)}}-Q_{{\text{SWCNTs}}^{\left(Pt-\text{SWCNTs}\right)}}$$

(5) $$\Delta Q_{Pt}=Q_{Pt^{\left(\text{gas}-Pt-\text{SWCNTs}\right)}}-Q_{Pt^{\left(Pt-\text{SWCNTs}\right)}}$$

### 3.1. SO_2_

SO_2_ is colorless, corrosive, and has a strong pungent odor. Moreover, when SO_2_ is dissolved in bodies of water, sulfurous acid rain is generated, which is harmful to the environment. SO_2_ can also form sulfuric acid when dissolved in water, which can irritate the mucous membrane of the eyes and nose.

**Figure 2.**
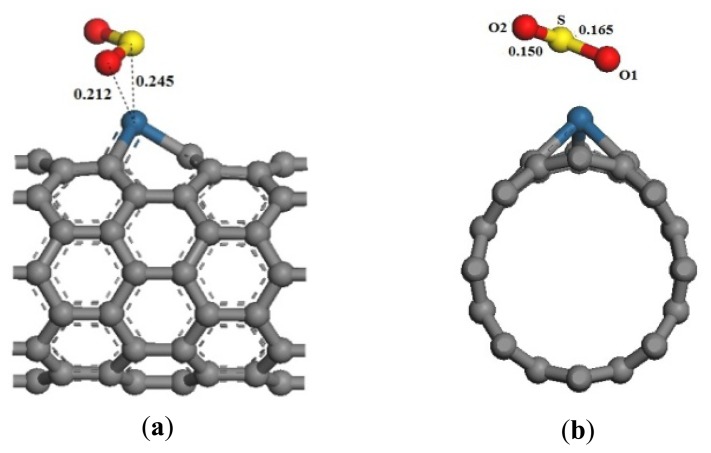
Structural model of the SO_2_-Pt-SWCNT adsorptive system. (**a**) Front view; (**b**) side view.

The full geometric optimization of the Pt-SWCNTs and SO_2_ adsorption model is shown in Figure 2. An oxygen atom O1 points to Pt, with Pt-O1 and Pt-S distances of 0.212 and 0.245 nm, respectively. The reaction adsorption energy is –1.225 eV (Table 1), which denotes an exothermic and spontaneous reaction. By contrast, the reaction adsorption energy of intrinsic SWCNTs is –0.830 eV, so Pt-doping enhances the interaction between SO_2_ and SWCNTs. Pt is not only a sensing element of Pt-SWCNTs, but also an active site. Strong interaction with gas molecules adsorbed on the surface results in deformation of Pt-SWCNTs and elongation of the Pt-C bond.

The frontier orbital energy difference of SO_2_ and Pt-SWCNTs is *E_H-L_* ≪ *E_L-H_*. A Pt-SWCNT electron only needs to overcome a 0.158 eV energy barrier to transfer to SO_2_, whereas a SO_2_ electron needs to overcome a 3.818 eV energy barrier to transfer to Pt-SWCNTs. Therefore, Pt-SWCNTs provide electrons to SO_2_ in the adsorption process. A portion of electrons fill the anti-bonding orbital of S-O1, changing the bond length from 0.143 nm to 0.165 nm. O2 is far from the CNT surface, so the interaction is small, allowing only a small change in the bond length of S-O2 (0.150 nm).

According to the respective Mulliken charge populations, SWCNTs of Pt-SWCNTs have 0.147 positive charge and Pt has 0.147 negative charge before adsorption. After the adsorption process, SWCNTs have 0.509 positive charge, whereas Pt has 0.116 negative charge. SO_2_ obtains 0.393 electrons during the adsorption reaction with Pt-SWCNTs, which is 4.6 times than intrinsic SWCNTs (Table 2). Charge variation (Δ*Q_SWCNTs_*, Δ*Q_Pt_*) of SWCNTs and Pt are 0.362 and 0.031, respectively (Table 3). Therefore, SO_2_ obtains electrons mainly from SWCNTs, whereas the Pt exhibits a small charge change.

**Figure 3.**
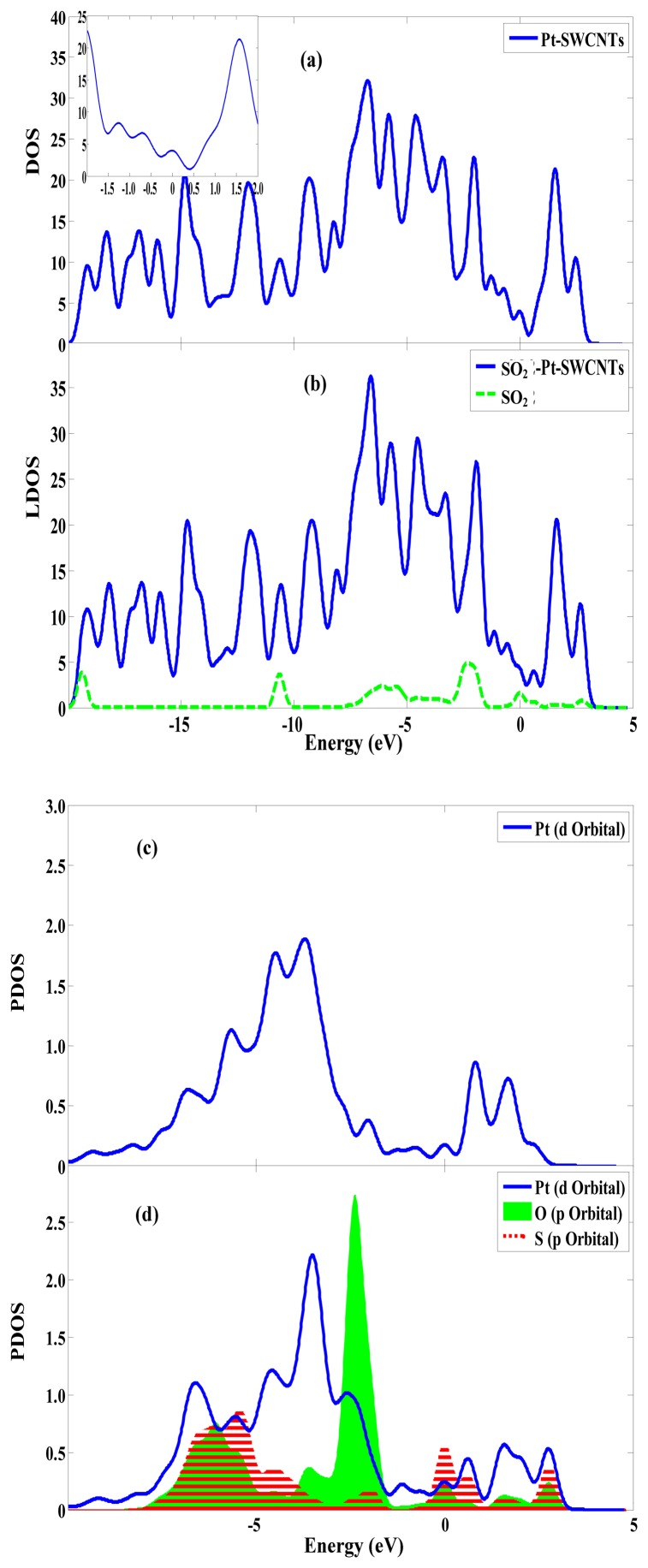
The density of states of Pt-SWCNTs before and after SO_2_ adsorption. (**a**) The DOS of Pt-SWCNTs; (**b**) The LDOS of SO_2_-Pt-SWCNTs and SO_2_ in SO_2_-Pt-SWCNTs; (**c**) The PDOS of Pt in Pt-SWCNTs; (**d**) The PDOS of Pt, O and S in SO_2_-Pt-SWCNTs. (Fermi level is 0eV).

**Figure 4.**
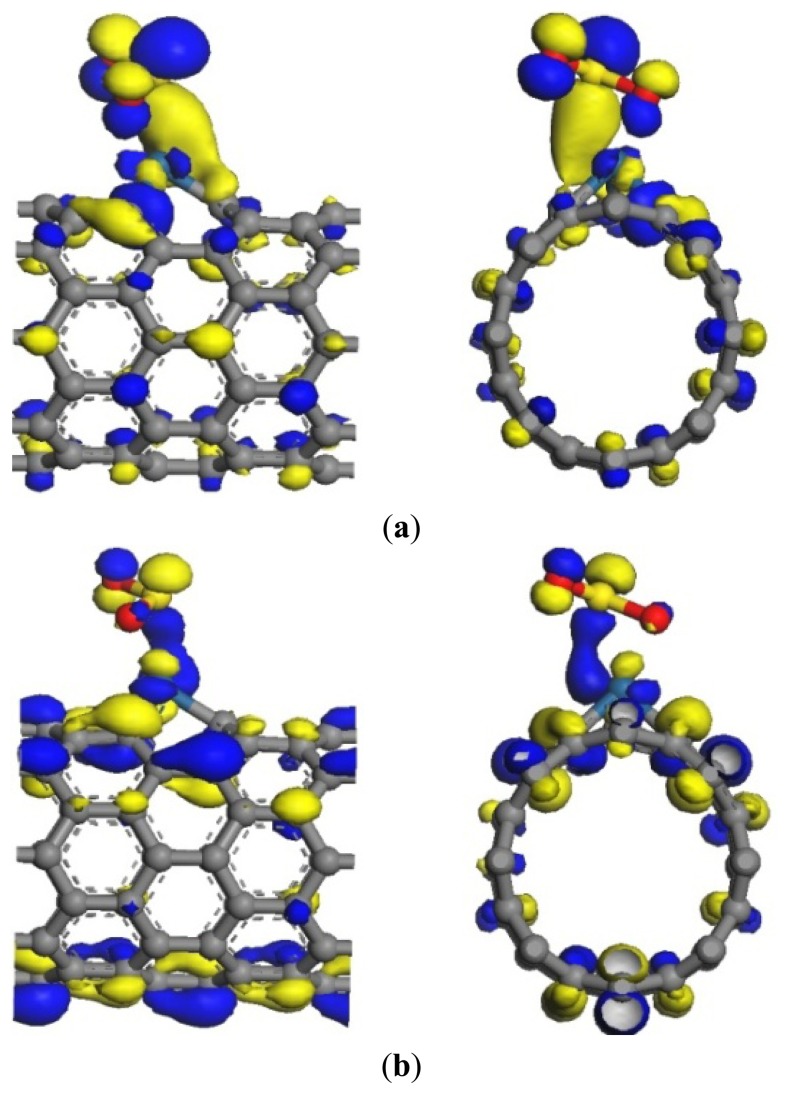
Frontier orbital energy level of SO_2_-Pt-SWCNTs. (**a**) HOMO (–5.208 eV); (**b**) LUMO (–4.923 eV).

The transfer of a large number of electrons during adsorption causes the redistribution of system charges. The density of states (DOS) near the Fermi level appears to be impure, for example there is a peak in –0.5eV. And the DOS between HOMO and LUMO changes. Figure 3 shows that these impure states are caused by SO_2_ adsorption. The *p* orbitals of S and O atoms have a large overlap with the *d* orbitals of Pt atom, and it demonstrates that SO_2_ can strongly hybridize with Pt [23]. This has significant effect on the frontier orbital of the adsorption system, which changes the HOMO and LUMO orbital formation, causing the change in the DOS. Figure 4a shows that the *p* orbitals of C1 and C3 form *σ* bond with S. In Figure 4b, the *d* orbitals of Pt and the *p* orbitals of S are hybridized. The frontier orbital energy gap *E*_g_ of the system is 0.285 eV after adsorbing SO_2_, which is reduced by 0.047 eV compared with that in the non-adsorbed SO_2_. This is beneficial for the transfer of electrons between HOMO and LUMO, thereby enhancing conductivity.

SO_2_ adsorption on the surface of Pt-SWCNTs has large adsorption energy and can form a stable structure. The *p*-type Pt-SWCNTs [11] donate electrons and increase the number of hole carriers, reducing the frontier orbital energy, diminishing energy gap *E*_g_, and enhancing conductivity. Pt-SWCNTs are highly responsive to SO_2_, the doped Pt effectively improved the adsorption sensitivity of SWCNTs to SO_2_.

### 3.2. H_2_S

H_2_S which is the simplest hydride of sulfur, is a colorless toxic gas that smells like rotten eggs and is strongly corrosive. It is also harmful to human health. The S in H_2_S is at the lowest valence state, so it is strongly reducible.

**Figure 5.**
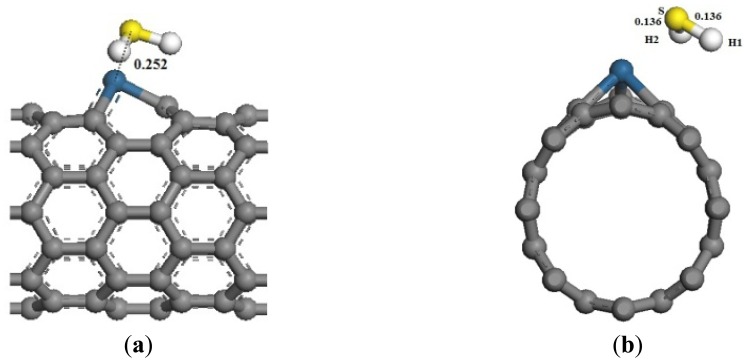
Structural model of H_2_S-Pt-SWCNT adsorptive system. (**a**) Front view; (**b**) side view.

**Figure 6.**
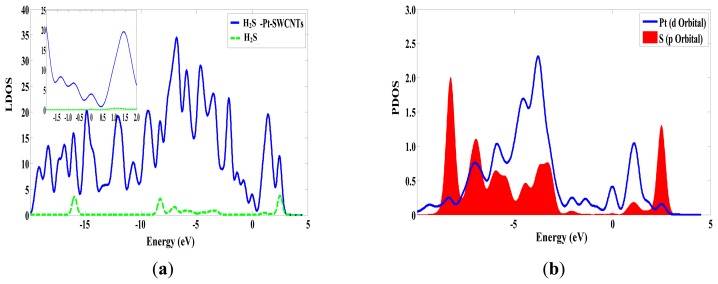
The density of states of H_2_S-Pt-SWCNTs. (**a**) The DOS of H_2_S-Pt-SWCNTs and the LDOS of H_2_S in H_2_S-Pt-SWCNTs. (**b**) The PDOS of Pt and S in H_2_S-Pt-SWCNTs.

**Figure 7.**
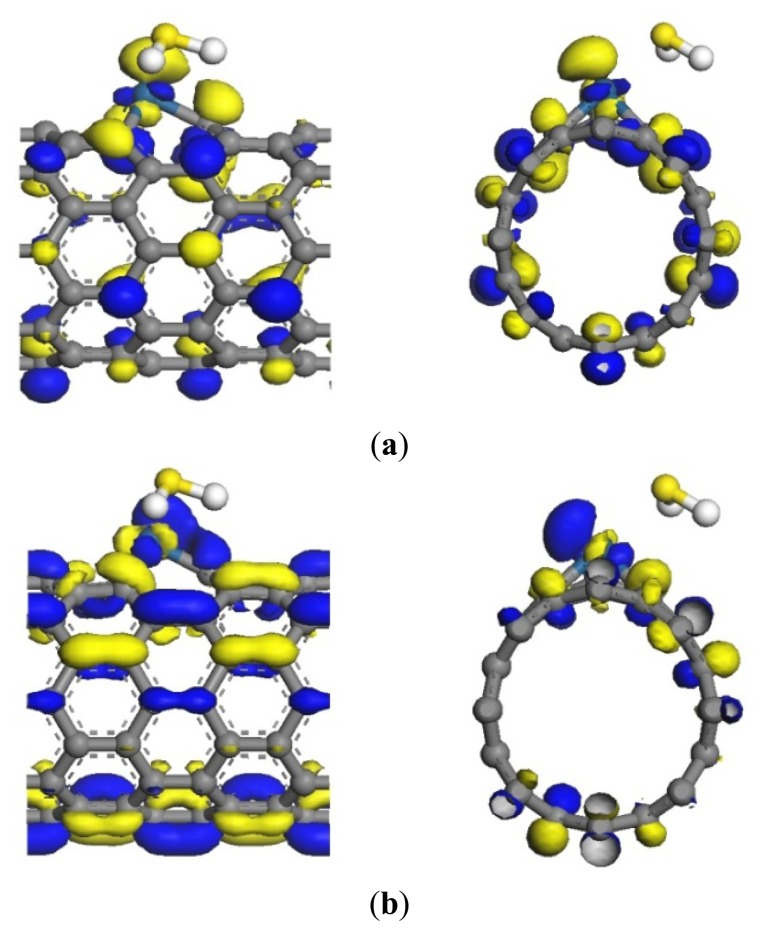
Frontier orbital energy level of H_2_S-Pt-SWCNTs. (**a**) HOMO (–4.425 eV); (**b**) LUMO (–4.142 eV).

The adsorption reaction of Pt-SWCNTs and H_2_S is also exothermic, with *E_b_* of –0.977 eV, more than intrinsic SWCNTs (–0.591eV in Table 2). The frontier orbital energy differences are *E_H-L_* = 4.438 eV and *E_L-H_* = 1.519 eV, therefore H_2_S provides electrons to Pt-SWCNTs in this reaction. Mulliken charge analysis (Table 3) shows that H_2_S donates 0.285 electrons almost 22 times more than intrinsic SWCNTs. Pt and SWCNTs have 0.019 and 0.266 electrons, respectively, after H_2_S is adsorbed on Pt-SWCNTs (Figure 5). A large number of electron transformations convert Pt-SWCNTs from *p*-type to *n*-type. *E_g_* of the adsorption system is 0.283 eV, which is reduced by 0.049 eV compared that with non-adsorbed H_2_S, thus enhancing conductivity. H_2_S-Pt-SWCNT frontier orbitals concentrate on Pt-SWCNTs, and H_2_S is not involved in the composition of HOMO and LUMO orbitals. Figure 6 shows that the DOS of H_2_S is not distributed between the HOMO and LUMO, and the DOS near the Fermi level is basically the same as that of Pt-SWCNTs, which is consistent with the results of frontier orbitals (Figure 7). The *p* orbitals of S have a large overlap with the *d* orbitals of Pt, and the strong interaction of them enhances the adsorption between H_2_S and the nanotube surface.

From the comparison results of adsorption energy and transfer charge, it can be seen that doped Pt obviously improves the adsorption ability of the instrinsic SWCNTs to H_2_S. H_2_S adsorbs on the surface of Pt-SWCNTs and donates substantial electrons to Pt-SWCNTs, which converts CNTs from *p*-type to *n*-type. The frontier orbital energy is increased, while the conductivity is enhanced because of the decrease in the frontier orbital energy gap.

### 3.3. CO

CO is a colorless, non-irritating gas. However when it enters the human body, CO combines with blood hemoglobin, which prevents the union of hemoglobin and oxygen, leading to body tissue hypoxia and even suffocation. The C atom in CO has +2 valence electrons and that can be further oxidized to +4. Accordingly, CO is a reducing gas that provides electrons in reactions.

**Figure 8.**
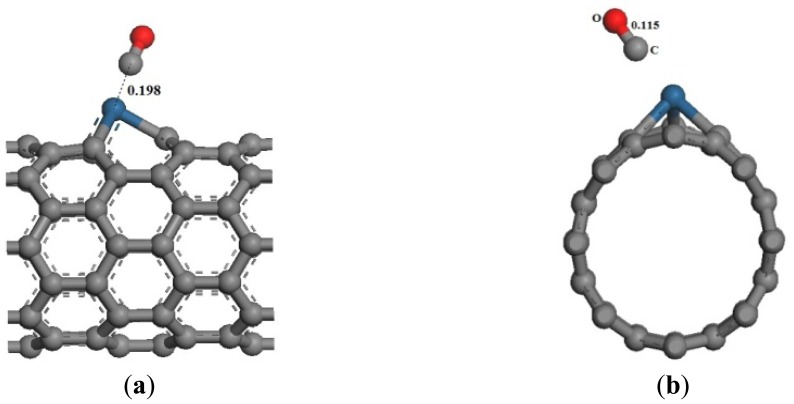
Structural model of CO-Pt-SWCNT adsorptive system. (**a**) Front view; (**b**) side view.

The optimized adsorption structure of CO adsorbed on Pt-SWCNTs is shown in Figure 8, which is consistent with reference [24], C atoms point to Pt, whereas O atoms point away from the CNT surface. The adsorption reaction is exothermic. High adsorption energy results in a tight bond between gases and CNTs, as well as an interaction distance of 0.198 nm.

**Figure 9.**
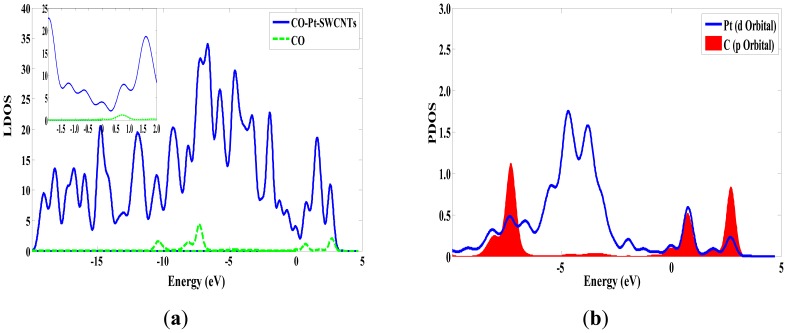
The density of states of CO-Pt-SWCNTs. (a) The DOS of CO-Pt-SWCNTs and the LDOS of CO in CO-Pt-SWCNTs. (b) The PDOS of Pt and C in CO-Pt-SWCNTs.

**Figure 10.**
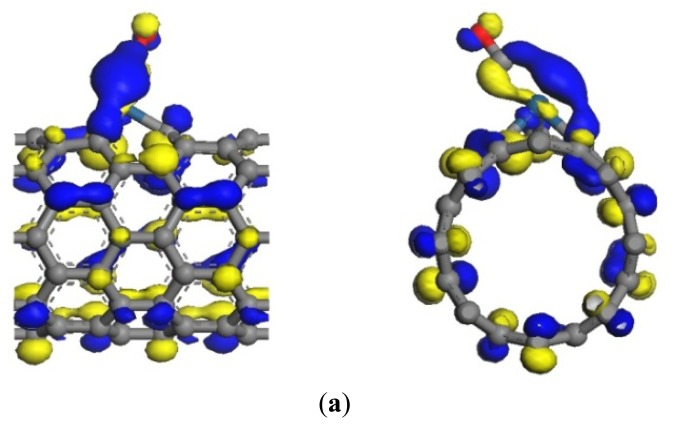

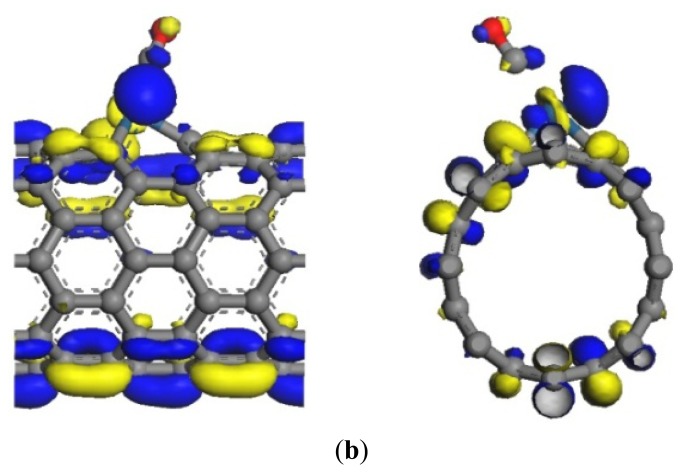
Frontier orbital energy level of CO-Pt-SWCNTs. (**a**) HOMO (–4.797 eV); (**b**) LUMO (–4.455 eV).

The same as the previous two gases, the adsorption energy and the transfer charge between Pt-SWCNTs and CO are increased obviously, enhancing the adsorption. Figure 9 shows that the *p* orbitals of C in CO have an overlap with the *d* orbitals of Pt, especially near the Fermi level. The DOS near the Fermi level is changed, and the peak at –7 eV is split, which is related to CO adsorption. The contributions of CO to HOMO and LUMO are mainly on the *p* orbitals of C and O atoms (Figure 10), which change the system configuration of frontier orbitals, changing the DOS of the system. During adsorption, CO provides 0.181 electrons (Table 3), *p*-type CNTs obtain electrons, and the number of hole carriers decreases. Frontier orbital energy and energy gap *E*_g_ increase, decreasing conductivity.

## 4. Discussion

Pt is a heavy metal with an atomic number of 78. Its outer core 5*d* orbitals have nine electrons and an unpaired *d* electron, so it easily absorbs electrons to reach a steady state. Pt doped into SWCNTs obtains electrons from C. In Pt-SWCNTs, Pt has 0.147 electrons, C1, C2, and C3 adjacent to Pt have 0.028, 0.029, and 0.097 electrons, respectively, which form an electron accumulation zone around the Pt atom. Given that Pt easily obtains electrons, when SO_2_ reacts with Pt-SWCNTs, SWCNTs donate most of the electrons, and a small charge change in Pt (Δ*Q_Pt_*) occurs. On the contrary, when the target gases are H_2_S and CO, Pt exhibits a large charge change.

## 5. Conclusions

In this study, the adsorptions of three gases on the surface of Pt-SWCNTs were calculated based on DFT. The gas-sensing properties of Pt-SWCNTs were assessed according to the changes in adsorption energy, geometric structure, and electronic structure during adsorption. The main conclusions are as follows:

1. The doped Pt effectively improves the adsorption sensitivity of intrinsic SWCNTs to the three kind of gases.
2. The adsorption energy of the reaction between Pt-SWCNTs and SO_2_ is large, and numerous electrons are transferred from CNTs to the target gases. The frontier orbital energies (*E_HOMO_* and *E_LUMO_*) and *E*_g_ are decreased, and the electrical conductivity of Pt-SWCNTs is enhanced. Pt-SWCNTs have high sensitivity to SO_2_.
3. H_2_S is reduced when reacted with Pt-SWCNTs. H_2_S provides a large number of electrons, converting CNTs from *p*-type to *n*-type. The frontier orbital energies are increased, whereas *E*_g_ is decreased, thereby enhancing conductivity.
4. When CO is adsorbed on Pt-SWCNTs, CO provides electrons to *p*-type CNTs, decreasing the number of hole carriers. The frontier orbital energies and *E*_g_ are increased, decreasing conductivity.

Results of the theoretical calculation show that Pt-SWCNTs can respond to the three gases. The electrical characteristics of Pt-SWCNTs show different degrees of changes after adsorption of the test gases. As SO_2_ is adsorbed on Pt-SWCNTs, the CNTs lose electrons, the number of hole carriers is increased, and conductivity is enhanced. As H_2_S is adsorbed on the surface of CNTs, Pt-SWCNTs receive a large number of electrons and transform from *p*-type into *n*-type. The conductivity of Pt-SWCNTs is also enhanced. Comparing their adsorption energies and charge transformations, the sensitivity to SO_2_ is higher than the sensitivity of Pt-SWCNTs to H_2_S. Moreover, CO is an electron-donor gas, which reduces hole carriers and weakens conductivity. Therefore, Pt-SWCNTs can be used to fabricate gas sensors in detecting SO_2_, H_2_S, and CO gases.

## Figures and Tables

**Table 1.** Adsorption energy and structural parameters of Pt-SWCNTs adsorption.

	**SO**_**2**_	**H**_**2**_**S**	**CO**
d_Pt-C1_/nm	0.205	0.200	0.205
d_Pt-C2_/nm	0.201	0.202	0.202
d_Pt-C3_/nm	0.196	0.192	0.194
*E_b_*/eV	–1.225	–0.977	–1.386

**Table 2.** Adsorption energy and charge transform of intrinsic SWCNTs adsorption.

	*E_b_***/eV**	*Q_gas_***/e**
SO_2_-SWCNTs	–0.830	–0.067
H_2_S-SWCNTs	–0.591	0.013
CO-SWCNTs	–0.157	0.006

**Table 3.** Electrical structure parameters of the adsorption structures.

**System**	**Q**_**SWCNTs**_**/e**	**Q**_**Pt**_**/e**	**Q**_**gas**_**/e**	**ΔQ**_**SWCNTs**_**/e**	**ΔQ**_**Pt**_**/e**
Pt-SWCNTs	0.147	–0.147			
SO_2_-Pt-SWCNTs	0.509	–0.116	–0.393	0.362	0.031
H_2_S-Pt-SWCNTs	–0.019	–0.266	0.285	–0.166	–0.119
CO-Pt-SWCNTs	0.166	–0.347	0.181	0.019	–0.200
